# Comparison of User-Oriented Information Services on the Websites of Large Hospitals in China and the United States: Cross-sectional Study

**DOI:** 10.2196/27392

**Published:** 2021-12-29

**Authors:** Yang Zhong, Wenjuan Tao, Yanlin Yang, Hao Wu, Weimin Li, Jin Wen

**Affiliations:** 1 Academic Affairs Office West China School of Medicine, West China Hospital Sichuan University Chengdu China; 2 Institute of Hospital Management West China Hospital Sichuan University Chengdu China; 3 President’s Office West China Hospital Sichuan University Chengdu China

**Keywords:** hospital websites, internet, information services, marketing mix, 7Ps, health care information services, hospital management, hospitals, patient services, eHealth

## Abstract

**Background:**

Many people use the internet to access health care information to support health care decisions, and hospital websites can be the first point of contact to provide health care information services for consumers. However, little is known about the current information services provided by the websites of large Chinese hospitals.

**Objective:**

The aim of this study is to evaluate and compare the information services of the websites of large hospitals in China and the United States. We hope that our findings will benefit hospital managers worldwide in providing service information on the web.

**Methods:**

This study adopted a cross-sectional analytical approach to evaluate the websites of large hospitals in China and the United States in 2020. A total of 300 large hospitals were randomly selected, of which half were in China and half were in the United States. Based on the 7Ps marketing mix, we identified 39 items that represent typical hospital website information services, covering the following seven dimensions: product, price, place, propagation, people, process, and physical evidence.

**Results:**

Most of the items (34/39, 87%) related to information services offered by hospital websites were less covered in China than in the United States; however, 5 items (appointments by a third-party platform, mobile payment, hospital value, hospital environment display, and physicians’ profiles) had higher coverage in China. The average scores for hospital websites in China and the United States were 13.25 (SD 2.99) points and 23.16 (SD 2.76) points, respectively. Generally, high scores were given to the south areas of China and north areas of the United States.

**Conclusions:**

Hospital websites in China lagged behind those in the United States with regard to information services offered. We recommend that hospital managers in China place more emphasis on the people, product, and propagation dimensions of the 7Ps marketing mix in the construction of information services on hospital websites. Through the comparison of the websites of large hospitals in China and the United States, our study findings can provide suggestions for forming standard hospital website construction guidelines worldwide.

## Introduction

The internet has become ubiquitous in people’s lives. As of October 2020, almost 5 billion people had direct access to the internet worldwide [1]. According to a report by the China Internet Network Information Center, as of March 2020, the penetration rate of internet users in China was 64.5%, which represents an increase of 4.9% when compared to this penetration rate in 2018 [2]. Most adults use the internet to access health care information to support health care decisions [3,4], and a hospital’s home page can serve as the first point of contact to provide health care information services for consumers. Thus, in the internet era, the hospital website has become an important window connecting various departments of a hospital with patients and society, as well as a fairly new marketing tool to current and potential customers [5]. Customers’ decision-making processes are often influenced by their perceptions and evaluations of a hospital’s website [6]. It is important for hospital websites to meet their expectations by providing useful and accessible information [7]. Therefore, the evaluation of hospital websites has become inevitable.

The subject of hospital website evaluation has been heavily researched in different studies, such as an analysis of private hospital websites promoting medical tourism in India, Malaysia, and Thailand [8]; an evaluation of the quality of private hospital websites in Turkey; an evaluation of top academic hospitals for finding endocrine surgeons [7]; an evaluation of website information provided by pediatric surgery centers in Australia and New Zealand [4]; and a ranking of children’s hospital websites in the United States [9]. However, previous studies have not fully examined the evaluation of websites of general or large hospitals. Various models or frameworks have been proposed to evaluate hospital websites, mainly focusing on the quality of the websites [10-12] or evaluating their accessibility [13]. Network engagement for hospitals is a skill that exists at the intersection of marketing and technical capability [6], and a well-designed hospital website that shows considerable service information could influence patients to take the first step into a facility. There is much less evidence of evaluating hospital websites from the perspective of information service.

Affected by the “Internet Plus Healthcare” strategy of the Chinese government (which proposes that internet technologies should be used to offer medical and public health services, promote family physician practices, improve drug supply and medical bill settlement, conduct medical education, and provide artificial intelligence services) [14], the overall construction level of hospital websites in China has improved significantly with time [15]. Furthermore, there is a substantial difference between past and modern hospital service concepts, especially in terms of offering user-oriented services and information. It is imperative to improve the information services provided by Chinese hospitals, especially those provided by large hospitals that offer intensive health care services. Therefore, it has become necessary to determine the current state of hospital websites in China. In addition, as shown in research that compared hospital websites from different countries [16], there is no study about the difference between hospital websites in China and other countries. The United States, where the internet emerged, was ranked third among countries with the most internet users after China, which had the highest number of internet users [17]. It would be interesting and meaningful to compare two powerful countries such as China and the United States, which have the largest economies in the world and represent a limited-income country and high-income country, respectively. There are 615,000 hospitals in the United States and 3,435,000 hospitals in China with a very large medical service market [18,19]. These hospitals could provide valuable information for global health care services when facing the increasing demand of the internet market.

This study aims to compare the information services of large hospital websites in China and the United States, based on the 7Ps marketing mix, and examine the problems that exist in Chinese hospital websites. We hope that our findings will benefit hospital managers not only in China and the United States but also around the world, especially in providing web-based service information.

## Methods

### Evaluation Framework Development

To the best of our knowledge, this study is the first to adopt the 7Ps marketing mix to form a logical evaluation framework for the sake of comparison, as this study focuses on the information services offered by hospital websites. The 7Ps marketing mix is a service marketing theory that was developed by Booms and Bitner [20] in 1981 by adding 3 new elements (people, process, and physical evidence) to the traditional marketing theory of 4Ps (product, price, place, and promotion). People, process, and physical evidence embody the characteristics of service marketing. Through a combination of production and consumption processes, this model allows customers to perceive high-quality service with the purpose of establishing, maintaining, and strengthening good long-term relationships with customers. According to the general definition and components of the 7Ps marketing mix, the corresponding definitions and components in relation to service information from hospital websites are presented in Multimedia Appendix 1. This evaluation framework consists of 7 dimensions and 39 items (components) for evaluating the service information of hospital websites. We consulted 10 hospital management or marketing experts to reach a consensus on determining and classifying the 39 items (components).

### Sampling

A large cross-sectional analysis approach was adopted to evaluate hospital websites in China and the United States. We randomly selected 150 large Chinese hospitals and 150 large American hospitals (a total of 300 hospitals) to conduct a survey of their website information services. Because it is very difficult and time-consuming to extract information from websites, we decided to randomly take 3 and 5 hospitals in each state and province to obtain 150 samples from the United States and China, respectively, after consulting with a statistician. All eligible hospitals (over 500 beds) from each administrative region of the two countries were included initially, and then a random sampling procedure was conducted. We focused on large hospitals because they usually serve more patients on average and their websites are visited by more users. Furthermore, large hospitals are more likely to represent the medical service capacity of a country. According to the current classification standards of Chinese hospitals, the definition of “large” refers to a Chinese hospital with at least 500 beds. Correspondingly, the same criterion was applied to the selection of American hospitals to map with the Chinese group. The number of beds for the selected hospitals in the United States was over 500 as well. The final sample of hospitals covered 31 provincial administrative regions in China (excluding Taiwan, Hong Kong, and Macao) and 45 states in the United States (excluding the states of Alaska, Montana, New Hampshire, Vermont, and Wyoming). Hospitals in Taiwan, Hong Kong, and Macao were excluded from the list of Chinese hospitals because their development and management systems are run differently from those in other regions of China for several historical reasons. Additionally, the population of these regions is too small to be included for explaining the general situation of China. We excluded 5 US states in our study because we failed to find hospitals with over 500 beds in these regions. Detailed information about the samples is provided in Multimedia Appendix 2.

### Data Collection

From February to June 2020, two independent reviewers identified the websites of the sampled hospitals using the Baidu and Google search engines. Each website was comprehensively assessed based on the 39 items of the previously developed evaluation framework to check if the corresponding information was available.

### Statistical Analysis

To create summarized scores of website service performance, the analytic method involved scoring the content according to 30 items, and each item was valued 1 point. The total score for each hospital website was reported in a range from 0 to 30, with a higher score on any given scale representing better comparative performance. The higher the score, the richer the information offered.

The 9 items that were not included in the scoring system were website accessibility, mobile payment, web account payment, appointment by telephone, appointment by official website, appointment by official app, appointment by a third-party platform, independent web page for hospital culture expressions, and patient-centered value. Website accessibility was not included in the scoring system because the information services and total scores for the websites could not be identified. Methods of making appointments and payments were excluded because there were no criteria showing which method was better. Independent web pages for hospital culture expressions and patient-centered value were not related to the availability of the service information offered but were related to how such information was offered by hospital websites; therefore, they were also left out of the scoring system.

Categorical variables were expressed as proportions and were tested using the chi-square test. Continuous variables were expressed as means and SDs and were tested by *t* test (if the sample observations followed a normal distribution and had homogeneity of variance) or Wilcoxon rank sum test (if the sample observations followed an abnormal distribution and had heterogeneity of variance). All analyses were conducted using Stata, version 12.0 (StataCorp LLC). All *P* values quoted below are 2-tailed and were considered significant when <.05.

## Results

Of the sampled hospitals, a total of 143 Chinese and 150 American hospital websites were included, because 7 Chinese hospitals had no website on the internet or the site could not be accessed during our research.

**Figure 1:**
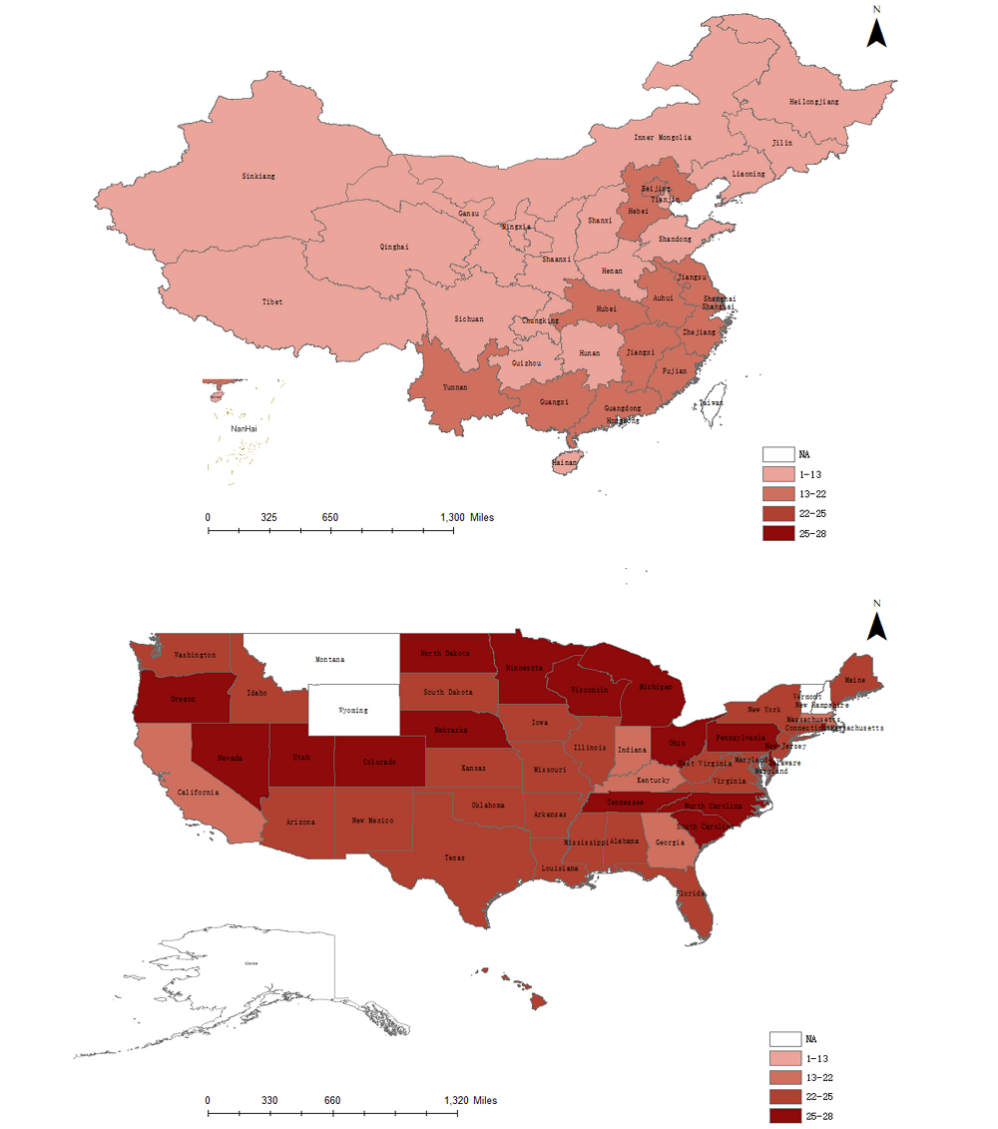
Comparison of the scores of hospital website information services in China and the United States.

All of the Chinese provinces/municipalities in this survey scored below 20 points, and more than 90% (29/31, 94%) scored in the range of 10 to 20 points. All of the studied American states scored above 20 points. As shown in Figure 1, there was a large gap between the two countries. It is interesting to note that the highest scores were generally given to the southern areas of China and northern areas of the United States. In terms of states or provinces/municipalities, the American hospital websites in Colorado, Delaware, and Rhode Island achieved the best scores (28 points); in China, the websites in Beijing, the capital city, obtained the highest score (17.2 points), followed closely by those in Zhejiang Province (17 points), Guangdong Province (15.43 points), and Shanghai Municipality (15.4 points). In the United States, hospitals in the state of California were rated the lowest (21.43 points); however, Sinkiang hospitals had the lowest score (5.4 points) in China.

**Figure 2:**
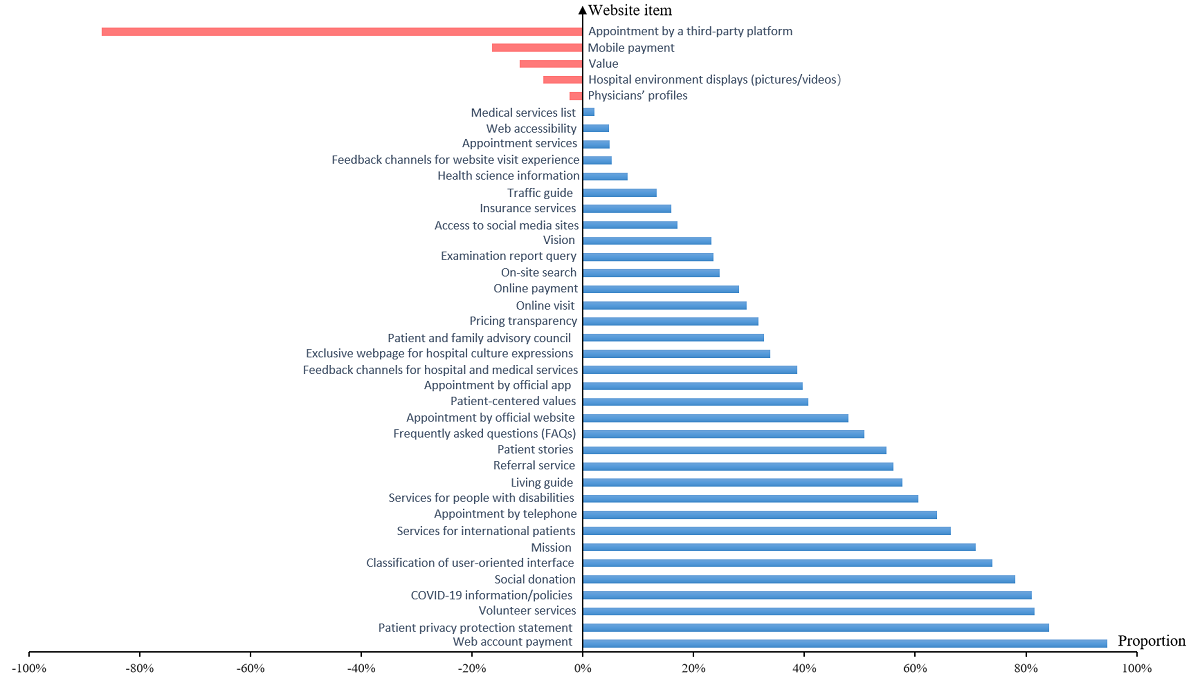
Differences in proportions of information service items on American and Chinese hospital websites. The proportion difference equals the percentage of accessibility of an item on American hospital websites minus the percentage of accessibility of an item on Chinese hospital websites.

The comparative results of 39 items representing the information services available on the websites between China and the United States are shown in Table 1. Overall, the information services offered by hospital websites in China were less covered than those offered in the United States, except for the following five items: appointment by a third-party platform, mobile payment, hospital value, hospital environment display (pictures/videos), and physicians’ profiles. The item with the highest availability on websites in China and the United States was the medical services list (139/143, 97.2%) and appointment services (150/150, 100%), respectively. There were no information services about 2 items—web account payment and patient and family advisory council—on the Chinese hospital websites, and no appointment service by a third-party platform was available on American hospital websites. The data in Figure 2 are derived from the proportion of American hospital websites minus that of Chinese hospital websites for each information service item. Among the items in which Chinese hospital websites lagged behind, the five with the largest gaps were web account payment, patient privacy protection statement, volunteer services, COVID-19 information/policies, and social donation (Figure 2). The differences were all statistically significant (*P*<.001; Table 1).

**Table 1:** Comparison of the available number of hospitals by information service items of hospital websites between China and the United States (N=300).

Dimension and items		China (n=143), n (%)	United States (n=150), n (%)	Chi-square (*df*)	*P* value
**Product**					
	Medical services list	139 (97.2)	149 (99.3)	1.98 (1)	.16
	Referral service	19 (13.3)	104 (69.3)	94.41 (1)	<.001
	Online visit	82 (59)	132 (88.6)	33.94 (1)	<.001
	Examination report query	94 (65.7)	134 (89.3)	23.62 (1)	<.001
	Insurance services	103 (72)	132 (88)	11.76 (1)	.001
	Living guide	8 (5.6)	95 (63.3)	107.06 (1)	<.001
	Services for people with disabilities	4 (2.8)	95 (63.3)	119.92 (1)	<.001
	Services for international patients	7 (4.9)	107 (71.3)	135.95 (1)	<.001
**Price**					
	Pricing transparency	66 (46.2)	116 (77.9)	30.24 (1)	<.001
	Online payment	95 (66.4)	142 (94.7)	37.75 (1)	<.001
	Mobile payment	95 (66.4)	75 (50)	8.12 (1)	.004
	Web account payment	0 (0)	141 (94.6)	N/A^a^	N/A
**Place**					
	Web accessibility	143 (95.3)	150 (100)	7.17 (1)	.007
	Traffic guide	121 (84.6)	147 (98)	16.8 (1)	<.001
	Appointment services	136 (95.1)	150 (100)	7.52 (1)	.006
	Appointment by telephone	43 (31.4)	143 (95.3)	128.38 (1)	<.001
	Appointment by official website	40 (29.2)	115 (77.2)	66.2 (1)	<.001
	Appointment by official app	20 (14.6)	81 (54.4)	49.4 (1)	<.001
	Appointment by a third-party platform	119 (86.9)	0 (0)	N/A	N/A
**Propagation**					
	Vision	64 (44.8)	102 (68)	16.11 (1)	<.001
	Mission	34 (23.8)	142 (94.7)	153.38 (1)	<.001
	Value	123 (86)	112 (74.7)	5.94 (1)	.02
	Exclusive web page for hospital culture expressions	85 (59.4)	140 (93.3)	47.19 (1)	<.001
	Patient-centered values	79 (55.2)	144 (96)	66.87 (1)	<.001
	Health science information	119 (83.2)	137 (91.3)	4.37 (1)	.04
	COVID-19 information/policies	13 (9.5)	135 (90.6)	188.08 (1)	<.001
	Patient stories	14 (9.9)	97 (64.7)	92.99 (1)	<.001
	Access to social media sites	109 (76.2)	140 (93.3)	16.79 (1)	<.001
**People**					
	Physicians’ profiles	136 (95.1)	139 (92.7)	0.75 (1)	.39
	Patient and family advisory council	0 (0)	49 (32.7)	56.09 (1)	<.001
	Patient privacy protection statement	16 (11.2)	143 (95.3)	208.85 (1)	<.001
	Volunteer services	15 (10.5)	138 (92)	194.94 (1)	<.001
	Social donation	13 (9.3)	131 (87.3)	176.45 (1)	<.001
	Feedback channels for hospital and medical services	73 (51.1)	131 (89.7)	52.05 (1)	<.001
	Feedback channels for website visit experience	5 (3.5)	13 (8.7)	3.39 (1)	.07
**Process**					
	Classification of user-oriented interface	22 (15.4)	134 (89.3)	160.81 (1)	<.001
	On-site search	101 (70.6)	143 (95.3)	32.08 (1)	<.001
	Frequently asked questions	15 (10.5)	92 (61.3)	81.63 (1)	<.001
**Physical evidence**					
	Hospital environment displays (pictures/videos）	138 (96.5)	134 (89.3)	5.66 (1)	.02

^a^N/A: not applicable.

Table 2 indicates the scoring results of information services available on hospital websites in China and the United States. The average scores for the 143 hospital websites in China and the 150 hospital websites in the United States were 13.25 (SD 2.99) points and 23.16 (SD 2.76) points, respectively. The scores of American hospital websites on 6 dimensions (product, price, place, propagation, people, and process) were all higher than those of Chinese hospital websites, and the differences were all statistically significant (*P*<.001). For the physical evidence dimension, namely, hospital environment display (pictures/videos), the score of the Chinese hospital websites was higher than that of the hospitals in the United States, and the difference was statistically significant (*z*=–2.37; *P*=.02). In addition, it can be seen from Table 2 that the largest gap existed in the people dimension, with a difference of more than 2 times in the average scores.

**Table 2:** Scoring results based on the 7Ps marketing mix for Chinese and American hospital websites.

Criterion	China (n=143)		United States (n=150)		*z* score	*P* value
	Score, mean (SD)	Score, P_50_ (P_25_, P_75_)^a^	Score, mean (SD)	Score, P_50_ (P_25_, P_75_)^a^		
Product (n=8)	3.22 (1.13)	3 (2, 4)	5.31 (1.37)	6 (5, 6)	11.00	<.001
Price (n=2)	1.13 (0.68)	1 (1, 2)	1.72 (0.49)	2 (1, 2)	7.79	<.001
Place (n=2)	1.80 (0.45)	2 (2, 2)	1.98 (0.14)	2 (2, 2)	4.64	<.001
Propagation (n=7)	3.33 (1.23)	3 (2, 4)	5.77 (1.16)	6 (5, 7)	12.46	<.001
People (n=7)	1.85 (0.80)	5 (5, 6)	5.03 (0.88)	2 (1, 2)	14.67	<.001
Process (n=3)^b^	0.97 (0.69)	1 (1, 1)	2.46 (0.65)	3 (2, 3)	19.13	<.001
Physical evidence (n=1)	0.97 (0.18)	1 (1, 1)	0.89 (0.31)	1 (1, 1)	–2.37	.02
Total (n=30)	13.25 (2.99)	13 (11, 15)	23.16 (2.76)	23 (22, 25)	14.43	<.001

^a^P_50_: 50th percentile; P_25_: 25th percentile; and P_75_: 75th percentile.

^a^Tested by a 2-tailed *t* test.

## Discussion

### Principal Findings

This is the first study to evaluate the website information services provided by large Chinese and American hospitals based on the 7Ps marketing mix. Overall, the hospital websites in the United States offer more information services compared to those in China, which suggests that US hospital managers consider this to be an important method of reaching their customers. According to the scores of the hospital websites, it can be clearly seen that the higher scores were mainly concentrated in the south and east regions of China, which have higher gross domestic products than those of other regions. This could be explained by the fact that these regions have more income and resources. It may also be the case that hospitals in these regions are more concerned about competition and have implemented a website as part of their strategy for attracting patients [21].

Specifically, the results suggest that large hospitals in China are largely lagging behind in offering user-oriented information services on their websites. Our findings revealed that the Chinese hospital websites focused primarily on basic services and information, such as the medical service list, appointment services, and physicians’ profiles, rather than paying more attention to the people dimension and involving the participation of and communication with the public, including patients, patients’ families, volunteers, social donors, and website users. Taking the item of the patient privacy protection statement as an example, the proportion difference between Chinese and American hospital websites was ranked first among the 30 comparable scoring items. This finding is consistent with a previous study, in which only 22% websites of leading Chinese general hospitals included a privacy and security policy or terms of use [22]. Similarly, a comparable study revealed that hospital websites in Kuwait focus primarily on promoting services provided by the hospital rather than on engaging and communicating with patients [23]; moreover, hospital websites in Italy function more as sources of information on admissions and services than as a means of communication between users and the hospital [24]. As for the product dimension, Chinese hospital websites are less thoughtful in caring about people with special needs, such as patients with disabilities or language communication barriers or people who require referral services or living guides.

For the propagation dimension, the function of disseminating public health information on websites is underused by large hospitals in China. At present, the COVID-19 pandemic is still active around the world; with the resumption of school and work, the Chinese government is implementing normalized epidemic prevention and control measures for a long-term fight against the disease. Hospitals in mainland China responded to the outbreak of COVID-19 positively by using information technology–enabled services [25]. Nonetheless, the number of hospital websites with COVID-19 prevention information and policies in the United States is approximately 10 times that of such websites in China. Additionally, the relatively low percentages we found for the items of vision, mission, and independent web pages for hospital culture expressions and patient-centered value on Chinese hospital websites indicate that there is significant potential for Chinese hospitals to promote their culture development.

Conversely, compared to the United States, our study shows that more Chinese hospital websites contain the following information services: appointment by a third-party platform, mobile payment, physicians’ profiles, hospital value, and environment display.

Two factors can be mainly attributed to the differences in appointments by third-party platforms and mobile payments between the two countries. First, the payment method for medical expenses in a country is closely related to the country’s medical insurance system. In the United States, large hospitals are often tied to (large) employers, such as Kaiser Permanente. Patients do not have the choice to “shop” for hospitals and may not wish to do so. This is also true of payment. Employees are often insured “for free” in the United States, as their employer provides them with insurance. People who are unemployed and have a low income are not insured, and they may have no money and no possibility to make mobile payments. In China, more than 95% of people are covered by basic health insurance [26]. Chinese people are usually free to make choices when seeking care and making payments.

Second, with the continuous development of mobile information technology, some basic functions performed by hospital websites in the past have been gradually transferred to mobile terminals, and the proportion of mobile medical services used in the process of patient medical treatment is increasing in China [27]. Chinese people, especially the younger generation, are more accustomed to using phones to make mobile payments—usually the consultation fee, which is a relatively small amount—to immediately confirm their appointments on the web. Mobile payment in China has become a life habit for Chinese people; this is not only reflected in the payment of medical expenses but also in all aspects of life because of its convenience and popularity. Chinese hospitals, under the influence of the “Internet Plus Healthcare” strategy, which is the application of internet technology in the medical industry and has been promoted by the Chinese government since its initiation in 2015, are also willing to cooperate with some large-scale application platforms, such as WeChat and Alipay, to implement these functions conveniently.

Therefore, Chinese users can easily obtain what they need without visiting hospital websites. In contrast, the relatively high rate of appointments on official websites and web account payments by American hospital websites has firmly locked American users into using them, and these websites have become the main source for Americans to obtain health information and services. With regard to the hospital values and environment display, as well as physicians’ profiles, we found in this study that incorporating hospital values, pictures of the hospital environment, and physicians’ profiles into the hospital’s introduction and home pages has almost become a fixed practice in most hospitals in China, which may partly explain the higher percentages of these services on Chinese hospital websites.

### Strengths and Weaknesses of This Study

By referring to the 7Ps marketing mix, we examined the websites’ information services in terms of the aspects of product, price, place, propagation, people, process, and physical evidence. To the best of our knowledge, no previous study has built evaluation items and thoroughly examined the information services of hospital websites based on the 7Ps marketing mix. In addition, this is the largest cross-sectional survey to date, assessing and comparing 150 hospital websites in China and 150 hospital websites in the United States.

This study may have several limitations that are important to note. On the one hand, we aimed to study the information services provided by hospital websites, which are different from traditional services; therefore, the classical 7Ps marketing mix may not be fully applicable to this study. However, we have adapted it to the websites’ information service features to perform a rational evaluation. On the other hand, we simply assigned 1 point to each item to build the scoring system for evaluating the information services of Chinese and American hospital websites; this approach may be unreasonable, as it does not take into account the weight of each item. Finally, there were 119 private (79.3%) and 31 public (20.7%) hospitals among the sample of 150 American hospitals. All 150 Chinese hospitals in the sample were public. We did not take the matter of public and private hospitals into account; moreover, the differences in social, political, legal, cultural, and other environmental factors between China and the United States were not considered, which could have affected the results.

### Potential Implications for Hospital Leaders

For hospital leaders, the findings of our study may have some potential implications based on the analysis of the product, price, place, propagation, people, process, and physical evidence dimensions of the 7Ps marketing mix.

#### Product Dimensions

It is necessary for hospital leaders to establish a user-friendly hospital website based on users’ actual needs. A study by Hakim and Deswindi [28] showed that the functional aspects of hospital websites are the most significant dimension because customers want to obtain in-depth information about a hospital’s organization, facilities, and list of services. Overall, it is necessary to reduce or categorize the information that has little relevance for website users; unfortunately, this problem is commonly found on the home pages of Chinese hospital websites. For example, Chinese hospital websites usually provide medical service information in the form of listings of departments; in contrast, most American hospital websites provide more detailed information about medical services, such as diseases, symptoms, departments, surgical operations, and other related services. This information is more helpful to web users who know little about their diseases or are not familiar with the related medical knowledge. For information services, it is important to strengthen the function of guidance rather than focusing on display only.

#### Price and Place Dimensions

With the development of mobile information technology, some important information services, such as payment and appointment methods, have been gradually transferred from websites to mobile terminals (eg, hospital apps and WeChat) in China. Therefore, customers no longer find it necessary to access a website to obtain required health information services. Mobile information technology has brought great convenience to people’s lives, and its use has become an inevitable trend. Compared to hospital websites in China, it was found that in the United States, a better balance is reached between the use of mobile devices and official websites. How to take advantage of mobile technology while also building a website that is valuable to visit has become an important issue that is worthy of consideration by Chinese hospital leaders.

#### Propagation Dimension

Culture development in an organization is a complicated and long-term process; however, one important measure is to clearly establish the mission of the organization, and this is especially urgent for Chinese hospital leaders given our findings. Furthermore, Chinese hospitals should make full use of the propagation function of their website platforms, whether to provide information on how to fight the pandemic or to disseminate public health knowledge. Another notable fact is that American hospital websites often use patient stories to build a positive image of their hospitals. This can readily enhance a customer’s feelings of trust toward a hospital. However, Chinese hospitals usually propagate information about the hospitals from the perspectives of medical staff skills or medical specialty strengths. As a result, because of information asymmetry between providers and patients, it is inevitable that certain obstacles in understanding will be created for ordinary users and will keep them at a distance.

#### People Dimension

The largest difference between Chinese and American hospitals lies in the people dimension, in which Chinese hospitals require much improvement to reach the level of US hospitals. According to our findings, Chinese hospitals should enhance their interactions on their websites with users and attach sufficient importance to social participation from individuals and other organizations. In detail, Chinese hospitals need to work more on feedback regarding hospital services and website visit experiences, develop volunteer services and social donation channels on their websites, show more respect to patients by emphasizing patient privacy protection statements, and attempt to learn more about patient and family advisory councils to determine if these organizations could work at Chinese hospitals.

#### Process Dimension

In the context of this research, the process dimension refers to the information or functions that are provided by a website to help users obtain the required information services. We investigated the situation of interface classification by users, site searches, and frequently asked questions pages of hospital websites between China and the United States, and we found that all of them were less covered on Chinese hospital websites. Therefore, more attention should be paid to the process dimension, as it directly reflects user-oriented ideas and is beneficial for improving website browsing experiences.

#### Physical Evidence Dimension

Vivid pictures or videos can be used to make websites more appealing to potential customers, so that they will spend more time on the websites. Although a few more Chinese hospitals displayed pictures or videos of the hospital environment according to our research, it was found that hospital websites in the United States as a whole are more aesthetic in terms of their visual design, which is worthy of learning by Chinese hospital administrators.

### Unanswered Questions and Future Research

In this paper, we studied the availability of information services on Chinese and American hospital websites; however, we did not investigate which factors could affect the performance of such websites. It would be interesting to know if there are certain correlations among some additional factors, such as the economic development level and population size of different regions in China and the United States, development level of informatization, and competition of medical services. This is worthy of analysis and discussion in further studies.

### Conclusions

Overall, hospital websites in China lagged behind their counterparts in the United States in providing user-oriented services and information. Most Chinese hospital websites focused primarily on basic services and information rather than involving the participation of and communication with the public. Additionally, culture development and the dissemination of public health knowledge need to be strengthened on Chinese hospital websites. Notably, we found that the transfer of basic services and information to mobile terminals has reduced the number of visits to hospital websites and may create obstacles to their further development. Hospital administrators can use the recommendations with respect to the product, price, place, propagation, people, process, and physical evidence dimensions to improve their hospitals’ websites. On the basis of the comparison of the large hospital websites in China and the United States, it is critical to develop standard website construction guidelines for hospitals worldwide.

## Appendix

Multimedia Appendix 1 7Ps marketing mix definition and components in relation to information services on hospital websites.

Multimedia Appendix 2 List of sample hospitals in China and the United States.
