# Supplementary material for: Comparison of User-Oriented Information Services on the Websites of Large Hospitals in China and the United States: Cross-sectional Study
Source: J Med Internet Res. 2021 Dec 29;23(12):e27392. doi: 10.2196/27392 (PMC8756340; doi:10.2196/27392)
Supplement: Multimedia Appendix 1 [file jmir_v23i12e27392_app1.docx]

**Multimedia Appendix 1. 7Ps marketing mix definition and components in relation to information services on hospital websites.**

| 7Ps Marketing mix | General definition and components of 7Ps Marketing mix | |  | 7Ps marketing mix definition and components in relation to information service from hospital website | |
| --- | --- | --- | --- | --- | --- |
|  | definition[^20^](#_ENREF_20) | components[^21^](#_ENREF_21) |  | definition | components |
| Product | A tangible physical good or an intangible service, or a blend of both | Service scope; Service quality; Service level; Service brand; Service content; Credit warranty; After-sale service |  | The hospital website provides medical service information and other information that meet the needs of users | Medical service list; Referral service; Online visit; Examination report query; Insurance services; Living guide; Services for the disabled; Services for the international patients |
| Price | The amount of money that is charged for the certain product | Discount and commission; Terms of payment; Customer perceived value; Match between brand name and price; Service differentiation |  | Website information about medical service prices and payment methods | Pricing transparency; Online payment (Mobile payment, Web Account Payment) |
| Place | The venue or location that provides products, which should be convenient for consumers to access | Location; Distribution channel; Closeness; Distribution scope |  | Website information about the user's access to medical services | Web accessibility; Traffic guide; Appointment services (Telephone, Official website, Official app, the Third-party platform) |
| Promotion Propagation | “Communicating information between the seller and potential buyers” through certain channels to encourage or persuade people to begin or to increase the consumption of products | Advertisement; Promotion; Human promotion; Public relation; Sale promotion |  | The hospital can build up the corporate image and spread health knowledge through public propaganda (website) | Vision, Mission, Value  (Independent webpage for hospital culture expressions, Patient-centered value); Health science information, COVID-19 information/policy; Patient stories; Access to social media sites |
| People | The personnel who participate in service delivery | Employee: training, personal judge, explanation capability, encouragement, appearance condition, interpersonal relationship; Attitude; Job quit rate; Recruitment |  | Including medical staff, patients and their families, website users, community members, etc. | Doctors' profile; Patient and family advisory council; Patient privacy protection statement; Volunteer services; Social donation; Feedback channels (hospitals and medical services); Feedback channels (website visit experience) |
| Processes | The procedures by which service is delivered | Service decision; Procedure; level of automation; Employee’s level of judgment; Guidance to the customer; Service process; After-sale service |  | Related information and functions provided by the website to help users obtain service information | Classification of user-oriented interface; On-site search; Frequently asked questions ((FAQs) |
| Physical evidence | The environment in which service is delivered | Environment: indoor decoration, color and arrangement; Equipment; Tangible product |  | Information about the hospital environment display on the website | Hospital environment display (pictures/videos) |
