# Supplementary material for: Comparison of User-Oriented Information Services on the Websites of Large Hospitals in China and the United States: Cross-sectional Study
Source: J Med Internet Res. 2021 Dec 29;23(12):e27392. doi: 10.2196/27392 (PMC8756340; doi:10.2196/27392)
Supplement: Multimedia Appendix 2 [file jmir_v23i12e27392_app2.docx]

**Multimedia Appendix 2.** List of sample hospitals in China and the United States.

| **No.** | **Country** | **Hospital name** | **Establishment** | **City** | **Province/State** | **Hospital Website** |
| --- | --- | --- | --- | --- | --- | --- |
| 1 | China | Xuanwu Hospital of Capital Medical University | 1958 | Beijing | Beijing | <https://www.xwhosp.com.cn/> |
| 2 | China | Beijing Tongren Hospital, Capital Medical University | 1886 | Beijing | Beijing | <http://www.trhos.com/> |
| 3 | China | Beijing University First Hospital | 1915 | Beijing | Beijing | <https://www.pkufh.com/> |
| 4 | China | Beijing Jishuitan Hospital | 1956 | Beijing | Beijing | <http://www.jst-hosp.com.cn/> |
| 5 | China | Peking Union Medical College Hospital, Chinese Academy of Medical Sciences | 1921 | Beijing | Beijing | <https://www.pumch.cn/index.html> |
| 6 | China | Tianjin First Central Hospital | 1942 | Tianjin | Tianjin | <http://www.tj-fch.com/> |
| 7 | China | Tianjin Medical University General Hospital | 1946 | Tianjin | Tianjin | <http://www.tjmugh.com.cn/> |
| 8 | China | Tianjin Huanhu Hospital | 1988 | Tianjin | Tianjin | <http://www.tnsi.org/> |
| 9 | China | Tianjin People's Hospital | 2004 | Tianjin | Tianjin | <http://www.umc.net.cn/> |
| 10 | China | Tianjin Third Central Hospital | 1957 | Tianjin | Tianjin | <http://www.tj3zx.cn/> |
| 11 | China | Cangzhou People's Hospital | 1961 | Cangzhou | Hebei | <http://www.czrmyy.com/> |
| 12 | China | Handan City First Hospital | 1950 | Handan | Hebei | <https://www.hdsdyyy.com/> |
| 13 | China | North China Petroleum Administration General Hospital | 1976 | Renqiu | Hebei | <http://www.hbsygljzyy.com/> |
| 14 | China | Tangshan People's Hospital | 1943 | Tangshan | Hebei | <http://www.tsrmyy.cn/> |
| 15 | China | Xingtai People's Hospital | 1945 | Xingtai | Hebei | <https://www.xtrmyy.cn/> |
| 16 | China | Taiyuan Central Hospital | 1958 | Taiyuan | Shanxi | <http://www.tyszxyy.com/web/zxyy/login> |
| 17 | China | The Second Hospital of Shanxi Medical University | 1919 | Taiyuan | Shanxi | <http://www.sydey.com/> |
| 18 | China | Linfen Central Hospital | 1950 | Linfen | Shanxi | [http://www.liNAench.com/index.html](http://www.linfench.com/index.html) |
| 19 | China | Yangquan First People's Hospital | 1948 | Yangquan | Shanxi | <http://www.yqsdyrmyy.com/> |
| 20 | China | The Third People's Hospital of Datong City | 1958 | Datong | Shanxi | <https://dtssyy.com/> |
| 21 | China | Ordos Central Hospital | 1950 | Ordos | Inner Mongolia | <http://www.ordoszxyy.org.cn/> |
| 22 | China | Inner Mongolia Baotou Steel Hospital | 1958 | Baotou | Inner Mongolia | <http://www.nmgbgyy.cn/> |
| 23 | China | The First Affiliated Hospital of Baotou Medical College, Inner Mongolia University of Science and Technology | 1957 | Baotou | Inner Mongolia | <http://www.nkdbyyfy.cn/> |
| 24 | China | Inner Mongolia Forestry General Hospital | 1956 | Yakeshi | Inner Mongolia | <http://www.nmglyzyy.com/> |
| 25 | China | Affiliated Hospital of Inner Mongolia University for Nationalities | 1972 | Tongliao | Inner Mongolia | <https://www.nmgmzdxfsyy.com/> |
| 26 | China | Anshan Central Hospital | 1924 | Anshan | Liaoning | <http://www.ashospital.com/> |
| 27 | China | Benxi Central Hospital | 1954 | Benxi | Liaoning | <http://www.bxszxyy.com/> |
| 28 | China | Xinhua Hospital Affiliated to Dalian University | 1953 | Dalian | Liaoning | <http://xhyy.dlu.edu.cn/> |
| 29 | China | Dalian Third People's Hospital | 1951 | Dalian | Liaoning | <http://www.dl3y.com/> |
| 30 | China | The First Affiliated Hospital of Dalian Medical University | 1930 | Dalian | Liaoning | <http://www.dmu-1.com/> |
| 31 | China | Changchun Central Hospital | 1948 | Changchun | Jilin | <https://www.ccszxyy.cn/> |
| 32 | China | 465 Hospital of Jilin Medical College | 1954 | Jilin | Jilin | <http://www.jl465.com/> |
| 33 | China | Jilin Chemical Group Corporation General Hospital | 1958 | Jilin | Jilin | NA |
| 34 | China | Jilin Provincial People's Hospital | 1946 | Changchun | Jilin | <http://www.jlsrmyy.net/> |
| 35 | China | The Second Hospital of Jilin University | 1936 | Changchun | Jilin | <https://www.jdey.com.cn/> |
| 36 | China | Heilongjiang Forest Industry General Hospital | 1972 | Harbin | Heilongjiang | <http://www.sgzyy.org.cn/> |
| 37 | China | Harbin 242 Hospital | 1954 | Harbin | Heilongjiang | <http://www.hrb242.org.cn/> |
| 38 | China | Daqing People's Hospital | 2000 | Daqing | Heilongjiang | <https://www.dqsrmyy.com/> |
| 39 | China | Daqing Oilfield General Hospital | 1960 | Daqing | Heilongjiang | <http://www.first-hospital.com/> |
| 40 | China | The Fourth Affiliated Hospital of Harbin Medical University | 1902 | Harbin | Heilongjiang | <http://1809300272.pool3-site.yun300.cn/> |
| 41 | China | Huashan Hospital Affiliated to Fudan University | 1907 | Shanghai | Shanghai | <https://www.huashan.org.cn/> |
| 42 | China | Shanghai Tongji Hospital | 1991 | Shanghai | Shanghai | <https://www.tongjihospital.com.cn/> |
| 43 | China | Shanghai Tenth People's Hospital | 1910 | Shanghai | Shanghai | <https://www.shdsyy.com.cn/web/> |
| 44 | China | The Ninth People's Hospital Affiliated to Shanghai Jiaotong University School of Medicine | 1920 | Shanghai | Shanghai | <http://www.9hospital.com.cn/djy_web/html/djy/portal/index/index.htm> |
| 45 | China | Zhongshan Hospital, Fudan University | 1937 | Shanghai | Shanghai | <http://www.zs-hospital.sh.cn/> |
| 46 | China | Taizhou People's Hospital | 1917 | Taizhou | Jiangsu | <http://www.jstzhospital.com/> |
| 47 | China | Changzhou First People's Hospital | 1918 | Changzhou | Jiangsu | <http://www.czfph.com/default.asp> |
| 48 | China | Affiliated Hospital of Jiangsu University | 1936 | Zhenjiang | Jiangsu | <http://www.jdfy.cn/> |
| 49 | China | Lianyungang First People's Hospital | 1951 | Lianyungang | Jiangsu | <http://www.lygyy.com.cn/> |
| 50 | China | The Second Affiliated Hospital of Nanjing Medical University | 1951 | Nanjing | Jiangsu | <http://www.jsnydefy.com/> |
| 51 | China | First Affiliated Hospital of Zhejiang University School of Medicine | 1947 | Hangzhou | Zhejiang | <http://www.zy91.com/> |
| 52 | China | Shaoxing People's Hospital | 1942 | Shaoxing | Zhejiang | <http://www.312000.net/> |
| 53 | China | Hangzhou Sixth People's Hospital | 1937 | Hangzhou | Zhejiang | <http://www.xixih.net/> |
| 54 | China | Zhejiang Provincial People's Hospital | 1984 | Hangzhou | Zhejiang | <http://www.hospitalstar.com/> |
| 55 | China | Ningbo First Hospital | 1913 | Ningbo | Zhejiang | <http://www.nbdyyy.com/> |
| 56 | China | Three People's Hospital of Bengbu City | 1953 | Bengbu | Anhui | <http://www.ahbbsy.com/> |
| 57 | China | Chaohu Hospital Affiliated to Anhui Medical University | 1951 | Chaohu | Anhui | <http://www.aychfy.com/> |
| 58 | China | Anqing Municipal Hospital | 1938 | Anqing | Anhui | <http://www.aqslyy.com.cn/> |
| 59 | China | Tongling People's Hospital | 1953 | Tongling | Anhui | <http://www.tlhos.com/> |
| 60 | China | Liu'an People's Hospital | 1949 | Liuan | Anhui | <http://www.layy.cn/> |
| 61 | China | Fuzhou First Hospital | 1898 | Fuzhou | Fujian | <http://www.fzsdyyy.com/> |
| 62 | China | Union Hospital of Fujian Medical University | 1860 | Fuzhou | Fujian | <http://www.fjxiehe.com/> |
| 63 | China | Quanzhou First Hospital | 1936 | Quanzhou | Fujian | <http://www.qzdyyy.com/> |
| 64 | China | Nanping First Hospital | 1905 | Nanping | Fujian | <http://www.npsdyyy.com/> |
| 65 | China | The First Affiliated Hospital of Xiamen University | 1937 | Xiamen | Fujian | <http://www.xmfh.com.cn/> |
| 66 | China | Nanchang First Hospital | 1934 | Nanchang | Jiangxi | <http://www.ncsdyyy.com/> |
| 67 | China | Jiangxi Provincial People's Hospital | 1897 | Nanchang | Jiangxi | <http://www.jxsrmyy.cn/> |
| 68 | China | Second People's Hospital of Jingdezhen City | 1971 | Jingdezhen | Jiangxi | <http://www.jdzdeyy.com/> |
| 69 | China | Pingxiang City People's Hospital | 1928 | Pingxiang | Jiangxi | <https://www.pxsrmyy.cn/> |
| 70 | China | Fuzhou First People's Hospital | 1949 | Fuzhou | Jiangxi | <http://www.fzdyyy.com/> |
| 71 | China | Affiliated Hospital of Binzhou Medical University | 1977 | Bingzhou | Shandong | <http://www.byfy.cn/> |
| 72 | China | The First People's Hospital of Jining City | 1896 | Jining | Shandong | <http://www.jnrmyy.com/> |
| 73 | China | Yantai Yuhuangding Hospital | 1890 | Yantai | Shandong | <https://www.ytyhdyy.com/> |
| 74 | China | Qilu Hospital of Shandong University | 1890 | Jinan | Shandong | <http://www.qiluhospital.com/> |
| 75 | China | Linyi People's Hospital | 1891 | Linyi | Shandong | <http://www.ly120.cn/> |
| 76 | China | The First Affiliated Hospital of Xinxiang Medical College | 1896 | Weihui | Henan | <https://www.xyyfy.com/> |
| 77 | China | Pingdingshan First People's Hospital | 1956 | Pingdingshan | Henan | <http://www.pdsdyrmyy.com/> |
| 78 | China | Henan Provincial People's Hospital | 1904 | Zhenzhou | Henan | <https://www.hnsrmyy.net/> |
| 79 | China | Anyang People's Hospital | 1887 | Anyang | Henan | <http://www.no1ayyy.com/> |
| 80 | China | The Fifth Affiliated Hospital of Zhengzhou University | 1915 | Zhenzhou | Henan | <http://www.ztzy.com/> |
| 81 | China | Wuhan Central Hospital | 1880 | Wuhan | Hubei | <http://www.zxhospital.com/> |
| 82 | China | Jingzhou First People's Hospital | 1949 | Jingzhou | Hubei | <http://www.jzyy1949.com/user_index.action> |
| 83 | China | Dongfeng Motor Corporation General Hospital | 1927 | Shiyan | Hubei | <https://www.dfmhp.com.cn/> |
| 84 | China | Wuhan University People's Hospital | 1923 | Wuhan | Hubei | <http://www.rmhospital.com/> |
| 85 | China | Hubei Jianghan Oilfield General Hospital | 1962 | Qianjiang | Hubei | <http://www.hbjhytzyy.com/> |
| 86 | China | Yongzhou Central Hospital | 1905 | Yongzhou | Hunan | <https://www.hnsyzszxyy.com/> |
| 87 | China | The First People's Hospital of Chenzhou City | 1907 | Chenzhou | Hunan | <http://www.czhospital.com/> |
| 88 | China | Hunan Provincial People's Hospital | 1912 | Changsha | Hunan | <http://www.hnsrmyy.com/> |
| 89 | China | The Second Affiliated Hospital of Nanhua University | 1949 | Hengyang | Hunan | <http://www.nh2h.com/> |
| 90 | China | Zhuzhou First Hospital | 1953 | Zhuzhou | Hunan | <http://www.zzszxyy.cn/> |
| 91 | China | Union Shenzhen Hospital of Huazhong University of Science and Technology | 1946 | Shenzhen | Guangdong | <https://www.sznsyy.net/> |
| 92 | China | Zhongshan People's Hospital | 1950 | Zhongshan | Guangdong | <http://www.zsph.com/> |
| 93 | China | Qingyuan People's Hospital | 1939 | Qingyuan | Guangdong | <http://www.qyry.com/> |
| 94 | China | Peking University Shenzhen Hospital | 1999 | Shenzhen | Guangdong | <https://www.pkuszh.com/> |
| 95 | China | The Second Affiliated Hospital of Guangzhou Medical University | 1982 | Guangzhou | Guangdong | <https://www.gyey.com/cn/index.aspx> |
| 96 | China | Shantou Central Hospital | 1922 | Shantou | Guangdong | <http://www.sthospital.com/> |
| 97 | China | Southern Hospital of Southern Medical University | 1941 | Guangzhou | Guangdong | [http://www.NAyy.com/](http://www.nfyy.com/) |
| 98 | China | Nanxishan Hospital of Guangxi Zhuang Autonomous Region | 1968 | Guilin | Guangxi | <http://www.nxsyy.com/> |
| 99 | China | People's Hospital of Guangxi Zhuang Autonomous Region | 1941 | Nanning | Guangxi | <http://www.gxhospital.com/> |
| 100 | China | Guilin People's Hospital | 1946 | Guilin | Guangxi | <http://www.glrmyy.com/> |
| 101 | China | Wuzhou Workers Hospital | 1903 | Wuzhou | Guangxi | <http://www.gr-hospital.com/> |
| 102 | China | The First Affiliated Hospital of Guangxi University of Science and Technology | 1933 | Liuzhou | Guangxi | <http://www.lyyfy.com/> |
| 103 | China | Third People's Hospital of Hainan Province | 1962 | Sanya | Hainan | <http://nk.sy.hn/> |
| 104 | China | Hainan Provincial Nongken General Hospital | 1952 | Haikou | Hainan | <http://www.nkhospital.com.cn/index.html> |
| 105 | China | The First Affiliated Hospital of Hainan Medical College | 1973 | Haikou | Hainan | <http://www.hyfyuan.com/> |
| 106 | China | Haikou People's Hospital | 1901 | Haikou | Hainan | <http://www.haikoumh.com.cn/> |
| 107 | China | Hainan Provincial People's Hospital | 1881 | Haikou | Hainan | <http://www.phhp.com.cn/> |
| 108 | China | The First Affiliated Hospital of Chongqing Medical University | 1957 | Chongqing | Chongqing | <http://www.hospital-cqmu.com/index.php> |
| 109 | China | The Second Affiliated Hospital of Chongqing Medical University | 1892 | Chongqing | Chongqing | <http://www.sahcqmu.com/> |
| 110 | China | Chongqing Fuling Central Hospital | 1914 | Chongqing | Chongqing | <http://www.old.cqflzxyy.com.cn/> |
| 111 | China | Chongqing Ninth People's Hospital | 1939 | Chongqing | Chongqing | <http://www.cq9yuan.com/> |
| 112 | China | Chongqing Three Gorges Central Hospital | 1929 | Chongqing | Chongqing | <http://www.cqsxzxyy.com/> |
| 113 | China | Ya'an People's Hospital | 1904 | Ya'an | Sichuan | <http://www.yasyy.com/> |
| 114 | China | Panzhihua Central Hospital | 1965 | Panzhihua | Sichuan | NA |
| 115 | China | Affiliated Hospital of Southwest Medical University | 1950 | Luzhou | Sichuan | <http://web.ahswmu.cn/> |
| 116 | China | Sichuan Provincial People's Hospital | 1941 | Chengdu | Sichuan | <https://www.samsph.com/> |
| 117 | China | Yibin First People's Hospital | 1903 | Yibin | Sichuan | <http://www.yb999.com/> |
| 118 | China | Guiyang First People's Hospital | 1919 | Guiyang | Guizhou | <http://www.gy1y.cn/> |
| 119 | China | Tongren People's Hospital | 1909 | Tongren | Guizhou | <http://www.trsrmyy.cn/> |
| 120 | China | Affiliated Hospital of Zunyi Medical University | 1969 | Zunyi | Guizhou | <http://www.zmchospital.com.cn/> |
| 121 | China | Guiyang Second People's Hospital | 1937 | Guiyang | Guizhou | <http://jyhosp.cn/> |
| 122 | China | Qianxinan Prefecture People's Hospital | 1967 | Xingyi | Guizhou | <http://www.qxnzrmyy.com/> |
| 123 | China | Yuxi City People's Hospital | 1950 | Yuxi | Yunnan | <http://www.yxhospital.com/> |
| 124 | China | Yuxi Traditional Chinese Medicine Hospital | 1987 | Yuxi | Yunnan | <http://www.yxszyyy.cn/index.html> |
| 125 | China | The Second Affiliated Hospital of Kunming Medical University | 1952 | Kunming | Yunnan | <http://www.kyfey.com/> |
| 126 | China | The First Affiliated Hospital of Kunming Medical University | 1941 | Kunming | Yunnan | <http://www.ydyy.cn/> |
| 127 | China | The First People's Hospital of Yunnan Province | 1932 | Kunming | Yunnan | <http://www.ypfph.com/> |
| 128 | China | People's Hospital of Tibet Autonomous Region | 1952 | Lasa | Tibet | <http://www.tarph.com/> |
| 129 | China | Hanzhong Central Hospital | 1965 | Hanzhong | Shaanxi | <http://www.hzcch.com/> |
| 130 | China | Yan'an University Affiliated Hospital | 1950 | Yan'an | Shaanxi | <http://www.yauhosp.com/> |
| 131 | China | Xianyang Central Hospital | 1953 | Xianyang | Shaanxi | NA |
| 132 | China | The First Hospital of Yulin City | 1951 | Yulin | Shaanxi | <http://www.ylsdyyy.com/> |
| 133 | China | Xi'an First Hospital | 1941 | Xi'an | Shaanxi | <http://www.xadyyy.com/> |
| 134 | China | Jiuquan People's Hospital | 1951 | Jiuquan | Gansu | <http://www.jqsyy.cn/> |
| 135 | China | Gansu Provincial People's Hospital | 1950 | Lanzhou | Gansu | <http://www.gsyy.cn/> |
| 136 | China | Zhangye City People's Hospital | 1956 | Zhangye | Gansu | <http://www.zy120.com/> |
| 137 | China | Pingliang City People's Hospital | 1939 | Pingliang | Gansu | NA |
| 138 | China | Qingyang People's Hospital | 1949 | Qingyang | Gansu | <http://www.qysrmyy.com/> |
| 139 | China | Affiliated Hospital of Qinghai University | 1959 | Xining | Qinghai | <http://www.qhuah.com/> |
| 140 | China | Qinghai Red Cross Hospital | 1949 | Xining | Qinghai | <http://www.qhrch.com/> |
| 141 | China | Qinghai Tibetan Hospital | 1983 | Xining | Qinghai | <http://tibethosp.com/> |
| 142 | China | Qinghai Provincial People's Hospital | 1927 | Xining | Qinghai | <http://www.qhsrmyy.com/> |
| 143 | China | People's Hospital of Ningxia Hui Autonomous Region | 1934 | Yinchuan | Ningxia | <http://www.nxrmyy.com/> |
| 144 | China | General Hospital of Ningxia Medical University | 1935 | Yinchuan | Ningxia | <http://www.nyfy.com.cn/> |
| 145 | China | Yinchuan First People's Hospital | 1957 | Yinchuan | Ningxia | <http://www.yc1yy.com/> |
| 146 | China | Yili Kazakh Autonomous Prefecture Friendship Hospital | 1936 | Yining | Xinjiang | NA |
| 147 | China | The Second Affiliated Hospital of Xinjiang Medical University | 1954 | Urumchi | Xinjiang | NA |
| 148 | China | Xinjiang People's Hospital | 1934 | Urumchi | Xinjiang | <http://www.xjrmyy.com/> |
| 149 | China | Karamay Central Hospital | 1956 | Karamay | Xinjiang | <http://www.xslmed.net/> |
| 150 | China | Urumqi Friendship Hospital | 1948 | Urumchi | Xinjiang | NA |
| 151 | The US | Huntsville Hospital | 1895 | Huntsville | Alabama | [www.hhsys.org](http://www.hhsys.org) |
| 152 | The US | DCH Regional Medical Center | 1916 | Tuscaloosa | Alabama | <https://www.dchsystem.com/Default.aspx?page=our_facilities%2fdch_regional_medical_center> |
| 153 | The US | UAB Hospital | 1945 | Birmingham | Alabama | <https://www.uabmedicine.org/> |
| 154 | The US | Mobile INAirmary Medical Center | 1910 | Mobile | Alabama | [https://www.iNAirmaryhealth.org/hospitals/mobile-iNAirmary](https://www.inairmaryhealth.org/hospitals/mobile-iNAirmary) |
| 155 | The US | Baptist Health Medical Center -Little Rock | 1921 | Little Rock | Arkansas | <https://www.baptist-health.com/location/baptist-health-medical-center-little-rock/> |
| 156 | The US | Banner Desert Medical Center | 1999 | Mesa | Arizona | <https://www.bannerhealth.com/locations/mesa/banner-desert-medical-center> |
| 157 | The US | Saint Joseph's Westgate Medical Center | 1895 | Phoenix | Arizona | <https://www.dignityhealth.org/arizona/locations/westgate> |
| 158 | The US | Valleywise Health Medical Center | 1877 | Phoenix | Arizona | <https://valleywisehealth.org/> |
| 159 | The US | Banner - University Medical Center Phoenix | 1911 | Phoenix | Arizona | <https://www.bannerhealth.com/locations/phoenix/banner-university-medical-center-phoenix> |
| 160 | The US | Kaiser Permanente Zion Medical Center | 1945 | San Diego | California | <https://thrive.kaiserpermanente.org/care-near-you/southern-california/san-diego/locations/kaiser-permanente-zion-medical-center/> |
| 161 | The US | Cedars-Sinai Medical Center | 1902 | Los Angeles | California | <https://www.cedars-sinai.org/> |
| 162 | The US | Sharp Grossmont Hospital | 1944 | La Mesa | California | <https://www.sharp.com/hospitals/grossmont/> |
| 163 | The US | Community Regional Medical Center | 1897 | Fresno | California | <https://www.communitymedical.org/hospitals-facilities/Community-Regional-Medical-Center> |
| 164 | The US | Scripps Mercy Hospital San Diego | 1890 | San Diego | California | <https://www.scripps.org/locations/hospitals/scripps-mercy-hospital/scripps-mercy-hospital-san-diego?tab=overview> |
| 165 | The US | Los Angeles Medical Center | 1945 | Los Angeles | California | <https://thrive.kaiserpermanente.org/care-near-you/southern-california/los-angeles/locations/los-angeles-medical-center-2/> |
| 166 | The US | Southern California Hospital at Hollywood | NA | Hollywood | California | <https://www.sch-hollywood.com/> |
| 167 | The US | UCHealth Memorial Hospital North | 2012 | Colorado Springs | Colorado | <https://www.uchealth.org/locations/uchealth-memorial-hospital-north/> |
| 168 | The US | UCHealth University of Colorado Hospital | 1921 | Aurora | Colorado | <https://www.uchealth.org/locations/uchealth-university-of-colorado-hospital-uch/> |
| 169 | The US | Hartford Hospital | 1854 | Hartford | Connecticut | <https://hartfordhospital.org/> |
| 170 | The US | Saint Francis Hospital and Medical Center | 1897 | Hartford | Connecticut | <http://www.stfranciscare.org/hartford> |
| 171 | The US | Yale-New Haven Hospital | 1826 | New Haven | Connecticut | <https://www.ynhh.org/> |
| 172 | The US | Christiana Hospital | 1985 | Newark | Delaware | <https://christianacare.org/facilities/christianahospital/> |
| 173 | The US | UF Health Shands Florida | 1958 | Gainesville | Florida | <https://ufhealth.org/uf-health-shands-hospital> |
| 174 | The US | Tampa General Hospital | 1927 | Tampa | Florida | <https://www.tgh.org/> |
| 175 | The US | Mount Sinai Medical Center | 1949 | Miami Beach | Florida | <https://www.msmc.com/> |
| 176 | The US | Jackson Memorial Hospital | 1952 | Miami | Florida | <https://jacksonhealth.org/jackson-memorial/> |
| 177 | The US | North Shore Medical Center | 1953 | Miami | Florida | [www.northshoremedical.com/](http://www.northshoremedical.com/) |
| 178 | The US | Broward Health Medical Center | 1920 | Fort Lauderdale | Florida | [www.browardhealth.org/bhmc](http://www.browardhealth.org/bhmc) |
| 179 | The US | NCH Baker Hospital Downtown | 1956 | Naples | Florida | <https://www.nchmd.org/all-locations/locations/hospital-emergency/nch-baker-hospital-downtown> |
| 180 | The US | Plantation General Hospital | NA | Platation | Florida | <https://plantationgeneral.com/> |
| 181 | The US | Northeast Georggia Medical Center Gainesville | 1951 | Gainesville | Georgia | <https://www.nghs.com/locations/gainesville/> |
| 182 | The US | Wellstar Atlanta Medical Center | 1901 | Atlanta | Georgia | <https://www.wellstar.org/locations/pages/wellstar-atlanta-medical-center.aspx> |
| 183 | The US | Medical Center Navicent Health | 1994 | Macon | Georgia | <https://www.navicenthealth.org/> |
| 184 | The US | Northside Hospital Gwinnett | 1970 | Lawrenceville | Georgia | <https://www.gwinnettmedicalcenter.org/facilities/northside-hospital-gwinnett> |
| 185 | The US | Piedmont Atlanta Hospital | 1905 | Atlanta | Georgia | <https://www.piedmont.org/locations/piedmont-atlanta/pah-home> |
| 186 | The US | The Queen's Medical Center | 1859 | Honolulu | Hawaii | <https://www.queens.org/the-queens-medical-center/queens-medical-center> |
| 187 | The US | MercyOne Des Moines | 1831 | Des Moines | Iowa | <https://www.mercyone.org/> |
| 188 | The US | University of Iowa hospitals and Clinics | 1898 | Iowa City | Iowa | <https://uihc.org/> |
| 189 | The US | St. Luke's Boise Medical Center | 1902 | Boise | Idaho | <https://www.stlukesonline.org/communities-and-locations/facilities/hospitals-and-medical-centers/st-lukes-boise-medical-center> |
| 190 | The US | The University of Chicago Medical Center | 1927 | Chicago | Illinois | <https://www.uchicagomedicine.org/> |
| 191 | The US | Advocate Lutheran General Hospital | 1897 | Par Ridge | Illinois | <https://www.advocatehealth.com/luth/> |
| 192 | The US | Northwestern Memorial Hospital | 1972 | Chicago | Illinois | <https://www.nm.org/locations/northwestern-memorial-hospital> |
| 193 | The US | Evanston Hospital | 1891 | Evanston | Illinois | <https://www.northshore.org/locations/our-hospitals/evanston-hospital/> |
| 194 | The US | Parkview Regional Medical Center | 1995 | Fort Wayne | Indiana | <https://www.parkview.com/locations/parkview-regional-medical-center/parkview-regional-medical-center> |
| 195 | The US | IU Health Methodist University | 1997 | Indianapolis | Indiana | <https://iuhealth.org/find-locations/iu-health-methodist-hospital> |
| 196 | The US | Methodist Hospitals-Northlake Campus | NA | Gary | Indiana | <https://www.methodisthospitals.org/> |
| 197 | The US | Deaconess Midtown Hospital | 1899 | Evansville | Indiana | <https://www.deaconess.com/Deaconess-Midtown-Hospital> |
| 198 | The US | Ascension Via Christi St. Francis | 1995 | Wichita | Kansas | <https://www.viachristi.org/location/via-christi-hospital-st-francis> |
| 199 | The US | The University of Kansas Hospital | 1906 | Kansas City | Kansas | <https://www.kansashealthsystem.com/locations/the-university-of-kansas-hospital> |
| 200 | The US | Wesley Medical Center | 1912 | Wichita | Kansas | <https://www.healthgrades.com/hospital-directory/kansas-ks/wesley-medical-center-hgst99354176170123> |
| 201 | The US | Norton Hospital | 1886 | Louisville | Kentucky | <https://nortonhealthcare.com/> |
| 202 | The US | University of Kentucky Albert B Chandler Hospital | 1962 | Lexington | Kentucky | <https://ukhealthcare.uky.edu/hospitals-clinics/albert-b-chandler-hospital> |
| 203 | The US | Baptist Health Louisville | 1924 | Louisville | Kentucky | <https://www.baptisthealth.com/louisville/Pages/default.aspx> |
| 204 | The US | UofL Health-Jewish Hospital | 1905 | Louisville | Kentucky | <https://www.uoflhealthnetwork.org/uofl-health-jewish-hospital> |
| 205 | The US | Ochsner Medical Center | 1942 | New Orleans | Louisiana | <https://www.ochsner.org/locations/ochsner-medical-center> |
| 206 | The US | Tulane Medical Center | 1834 | New Orleans | Louisiana | <https://tulanehealthcare.com/> |
| 207 | The US | Willis-Knighton Medical Center | 1924 | Shreveport | Louisiana | <https://www.wkhs.com/locations/hospitals/willis-knighton-medical-center> |
| 208 | The US | Our Lady of the Lake Regional Medical Center | 1923 | Baton Rouge | Louisiana | <https://ololrmc.com/> |
| 209 | The US | Beth Israel Deaconess Medical Center | 1896 | Boston | Massachusetts | <https://www.bidmc.org/> |
| 210 | The US | Umass Memorial Medical Center - University Campus | 1871 | Worcester | Massachusetts | <https://www.umassmemorialhealthcare.org/umass-memorial-medical-center-15> |
| 211 | The US | Charlton Memorial Hospital | 1885 | Fall River | Massachusetts | <https://www.southcoast.org/locations/charlton-memorial-hospital/> |
| 212 | The US | Massachusetts General Hospital | 1811 | Boston | Massachusetts | <https://www.massgeneral.org/> |
| 213 | The US | The Johns Hopkins Hospital | 1889 | Baltimore | Maryland | <https://www.hopkinsmedicine.org/the_johns_hopkins_hospital/> |
| 214 | The US | University of Maryland Medical Center | 1823 | Baltimore | Maryland | <https://www.umms.org/ummc> |
| 215 | The US | Maine Medical Center | 1874 | Portland | Maine | <https://mainehealth.org/maine-medical-center> |
| 216 | The US | Beaumont Hospital, Troy | 1977 | Troy | Michigan | <https://www.beaumont.org/locations/beaumont-hospital-troy> |
| 217 | The US | Spectrum Health Butterworth Hospital | 1873 | Grand Rapids | Michigan | <https://www.spectrumhealth.org/locations/spectrum-health-hospitals-butterworth-hospital> |
| 218 | The US | Sparrow Hospital | 1896 | Lansing | Michigan | <https://www.sparrow.org/sparrowhospital> |
| 219 | The US | Ascension Saint John Hospital | 1952 | Detroit | Michigan | <https://healthcare.ascension.org/Locations/Michigan/MIDET/Detroit-Ascension-St-John-Hospital> |
| 220 | The US | Henry Ford Hospital | 1915 | Detroit | Michigan | <https://www.henryford.com/locations/henry-ford-hospital> |
| 221 | The US | Abbott Northwestern Hospital | 1882 | Minneapolis | Minnesota | <https://www.allinahealth.org/abbott-northwestern-hospital> |
| 222 | The US | M Health Fairview University of Minnesota Medical Center | 1997 | Minneapolis | Minnesota | <https://www.fairview.org/locations/university-of-minnesota-medical-center-east-bank-hospital> |
| 223 | The US | Mayo Clinic Hospital - Saint Mary's Campus | 1889 | Rochester | Minnesota | <https://www.mayoclinic.org/patient-visitor-guide/minnesota/campus-buildings-maps/mayo-clinic-hospital-saint-marys-campus> |
| 224 | The US | Mercy Hospital South | 1827 | Saint Louis | Missouri | <https://www.mercy.net/about/mission/expansion/st-anthonys/> |
| 225 | The US | Barnes-Jewish Hospital | 1902 | Saint Louis | Missouri | <https://www.barnesjewish.org/> |
| 226 | The US | University Hospital | 1956 | Columbia | Missouri | <https://www.muhealth.org/locations/university-hospital> |
| 227 | The US | Cox North Hospital | 1906 | Springfield | Missouri | <https://www.coxhealth.com/our-hospitals-and-clinics/our-locations/cox-north/> |
| 228 | The US | North Mississippi Medical Center - Tupelo | 1937 | Trupelo | Mississippi | <https://www.nmhs.net/locations/profile/north-mississippi-medical-center-tupelo/> |
| 229 | The US | Saint Dominic Hospital | 1946 | Jackson | Mississippi | [www.stdom.com](http://www.stdom.com/) |
| 230 | The US | University of Mississippi Medical Center | 1903 | Jackson | Mississippi | [www.umc.edu/](http://www.umc.edu/) |
| 231 | The US | UNC Medical Center | 1952 | Chapel Hill | North Carolina | [www.uncmedicalcenter.org/uncmc/](http://www.uncmedicalcenter.org/uncmc/) |
| 232 | The US | Mission Hospital - Memorial Campus | 1996 | Asheville | North Carolina | <https://missionhealth.org/member-hospitals/mission/> |
| 233 | The US | Carolinas Medical Center | 1943 | Charlotte | North Carolina | <https://atriumhealth.org/locations/carolinas-medical-center> |
| 234 | The US | WakeMed Raleigh Campus | 1961 | Raleigh | North Carolina | [www.wakemed.org/raleigh-campus](http://www.wakemed.org/raleigh-campus) |
| 235 | The US | Vidant Medical Center | 1923 | Greenville | North Carolina | <http://www.vidanthealth.com/Locations/Hospitals/Vidant-Medical-Center> |
| 236 | The US | Sanford Medical Center Fargo | 1908 | Fargo | North Dakota | [https://www.saNAordhealth.org/locations/saNAord-medical-center-fargo](https://www.sanaordhealth.org/locations/saNAord-medical-center-fargo) |
| 237 | The US | Bryan Medical Center - East Campus | 1926 | Lincoln | Nebraska | <https://www.bryanhealth.com/locations/bryan-east-campus/> |
| 238 | The US | The Nebraska Medical Center | 1869 | Omaha | Nebraska | [www.nebraskamed.com/](http://www.nebraskamed.com/) |
| 239 | The US | Jersey Shore University Medical Center | 1904 | Neptune | New Jersey | <https://www.jerseyshoreuniversitymedicalcenter.com/> |
| 240 | The US | Bergen New Bridge Medical Center | NA | Paramus | New Jersey | [www.newbridgehealth.org](http://www.newbridgehealth.org/) |
| 241 | The US | Virtua Voorhees Hospital | 2011 | Voorhees | New Jersey | <https://www.virtua.org/locations/voorhees-hospital> |
| 242 | The US | Cooper University Hospital | 1887 | Camden | New Jersey | <https://www.cooperhealth.org/locations/cooper-university-hospital-one-cooper-plaza> |
| 243 | The US | Morristown Medical Center | 1892 | Morristown | New Jersey | <https://www.atlantichealth.org/locations/hospitals/morristown-medical-center.html> |
| 244 | The US | Presbyterian Hospital | NA | Albuquerque | New Mexico | <https://presbyterian-hospital.phs.org/Pages/default.aspx> |
| 245 | The US | UNM Hospital | 1954 | Albuquerque | New Mexico | <https://hsc.unm.edu/health/locations/unm-hospital.html> |
| 246 | The US | Renown Regional Medical Center | 1876 | Reno | Nevada | <https://www.renown.org/locations/hospitals/regional-medical-center/> |
| 247 | The US | Sunrise Hospital & Medical Center | 1958 | Las Vegas | Nevada | <https://sunrisehospital.com/> |
| 248 | The US | Crouse Hospital | 1887 | Syracuse | New York | [www.crouse.org](http://www.crouse.org/) |
| 249 | The US | Jamaica Hospital Medical Center | 1891 | Jamaica | New York | [www.jamaicahospital.org/](http://www.jamaicahospital.org/) |
| 250 | The US | Stony Brook University Hospital | 1980 | Stony Brook | New York | [www.stonybrookmedicine.edu](http://www.stonybrookmedicine.edu/) |
| 251 | The US | North Shore University Hospital | 1953 | Manhasset | New York | <https://nsuh.northwell.edu/> |
| 252 | The US | BronxCare Dr. Martin Luther King, Jr. Health Center | 1890 | Bronx | New York | [www.bronxcare.org](http://www.bronxcare.org/) |
| 253 | The US | Strong Memorial Hospital | 1925 | Rochester | New York | <https://www.urmc.rochester.edu/strong-memorial.aspx/> |
| 254 | The US | Olean General Hospital | 1898 | Olean | New York | [www.ogh.org](http://www.ogh.org/) |
| 255 | The US | NYC Health + Hospitals Bellevue | 1736 | New York | New York | <https://www.nychealthandhospitals.org/bellevue/> |
| 256 | The US | Saint Francis Hospital | NA | Tulsa | Oklahoma | <https://www.saintfrancis.com/location/saint-francis-hospital> |
| 257 | The US | SSM Health Saint Anthony Hospital - Oklahoma City | 1894 | Oklahoma City | Oklahoma | <https://www.ssmhealth.com/locations/st-anthony-hospital> |
| 258 | The US | Oklahoma University Medical Center | 1943 | Oklahoma City | Oklahoma | [www.oumedicine.com/oumedicalcenter](http://www.oumedicine.com/oumedicalcenter) |
| 259 | The US | INTEGRIS Baptist Medical Center | 1995 | Oklahoma City | Oklahoma | <https://baptist.integrisok.com> |
| 260 | The US | Aultman Hospital | 1892 | Canton | Ohio | [www.aultman.org/](http://www.aultman.org/) |
| 261 | The US | Ohio State University Wexner Medical Center | 1834 | Columbus | Ohio | <https://wexnermedical.osu.edu> |
| 262 | The US | UH Cleveland Medical Center | 1868 | Cleveland | Ohio | <https://www.uhhospitals.org/locations/uh-cleveland-medical-center> |
| 263 | The US | ProMedica Toledo Hospital | 1874 | Toledo | Ohio | <https://www.promedica.org/toledo-hospital/pages/default.aspx> |
| 264 | The US | Miami Valley Hospital | 1890 | Dayton | Ohio | [www.miamivalleyhospital.org/](http://www.miamivalleyhospital.org/) |
| 265 | The US | Oregon Health & Science University Hospital | NA | Portland | Oregon | <https://www.ohsu.edu/visit> |
| 266 | The US | Reading Hospital | 1886 | West Reading | Pennsylvania | <https://towerhealth.org/locations/reading-hospital> |
| 267 | The US | WellSpan York Hospital | 1880 | York | Pennsylvania | <https://www.wellspan.org/offices-locations/wellspan-york-hospital/> |
| 268 | The US | Lehigh Valley Hospital - Cedar Crest | 1974 | Allentown | Pennsylvania | <https://www.lvhn.org/locations/lehigh-valley-hospital-cedar-crest> |
| 269 | The US | Abington Hospital - Jefferson Health | 1914 | Abington | Pennsylvania | <https://www.abingtonhealth.org/our-locations/abington-hospital/> |
| 270 | The US | Lancaster General Hospital | 1893 | Lancaster | Pennsylvania | <https://www.lancastergeneralhealth.org/patient-and-visitor-iNAormation/find-a-location/lancaster-general-hospital> |
| 271 | The US | Rhode Island Hospital | 1857 | Providence | Rhode Island | <https://www.rhodeislandhospital.org/> |
| 272 | The US | MUSC Health University Hospital | 1955 | Charleston | South Carolina | <https://muschealth.org/patients-visitors/about-us> |
| 273 | The US | Prisma Health Richland Hospital | 1892 | Columbia | South Carolina | <https://www.palmettohealth.org/locations-directions/hospitals/richland> |
| 274 | The US | McLeod Regional Medical Center | 1906 | Florence | South Carolina | <https://www.mcleodhealth.org/locations/mcleod-regional-medical-center-florence/> |
| 275 | The US | Avera McKennan Hospital & University Health Center | 1897 | Sloux Falls | South Dakota | <https://www.avera.org/locations/mckennan/> |
| 276 | The US | Johnson City Medical Center | 1911 | Johnson City | Tennessee | <https://www.balladhealth.org/hospitals/johnson-city-medical-center> |
| 277 | The US | TriStar Centennial Medical Center | NA | Nashville | Tennessee | <https://tristarcentennial.com/> |
| 278 | The US | University of Tennessee Medical Center | 1956 | Knoxville | Tennessee | <https://www.utmedicalcenter.org/> |
| 279 | The US | Erlanger Baroness Hospital | 1889 | Chattanooga | Tennessee | <https://www.erlanger.org/baroness-hospital/baroness-hospital> |
| 280 | The US | Parkland Hospital | 1894 | Dallas | Texas | <https://www.parklandhospital.com/> |
| 281 | The US | Baylor University Medical Center | 1903 | Dallas | Texas | <https://www.bswhealth.com/locations/dallas/Pages/default.aspx> |
| 282 | The US | The University of Texas Medical Branch Galveston Campus | 1891 | Galveston | Texas | <https://www.utmbhealth.com/locations/hospitals-campuses/galveston-campus> |
| 283 | The US | Baptist Medical Center | 1903 | San Antonio | Texas | <https://www.baptisthealthsystem.com/location/detail/baptist-medical-center> |
| 284 | The US | Ben Taub General Hospital | 1963 | Houston | Texas | <https://www.harrishealth.org/locations-hh/Pages/ben-taub.aspx> |
| 285 | The US | John Peter Smith Hospital | 1877 | Fort Worth | Texas | <https://www.jpshealthnet.org/> |
| 286 | The US | Medical City Dallas | 1974 | Dallas | Texas | <https://medicalcityhealthcare.com/locations/medical-city-dallas/> |
| 287 | The US | Intermountain Medical Center | 2007 | Murray | Utah | <https://intermountainhealthcare.org/locations/intermountain-medical-center/> |
| 288 | The US | University of Utah Hospital | 1965 | Salt Lake City | Utah | <https://healthcare.utah.edu/locations/hospital/> |
| 289 | The US | VCU Medical Center | 1838 | Richmond | Virginia | <https://www.vcuhealth.org/locations/location-details?&practice=10> |
| 290 | The US | Carilion Roanoke Memorial Hospital | 1899 | Roanoke | Virginia | <https://www.carilionclinic.org/locations/carilion-roanoke-memorial-hospital> |
| 291 | The US | Henrico Doctors' Hospital | 1974 | Richmond | Virginia | <https://henricodoctors.com/> |
| 292 | The US | Sentara Norfolk General Hospital | 1888 | Norfolk | Virginia | <https://www.sentara.com/hampton-roads-virginia/hospitalslocations/locations/sentara-norfolk-general-hospital.aspx> |
| 293 | The US | Providence Regional Medical Center Everett Colby Campus | 1856 | Everett | Washington | <http://washington.providence.org/locations-directory/r/regional-medical-center> |
| 294 | The US | Providence Sacred Heart Medical Center and Children's Hospital | 1856 | Spokane | Washington | <http://washington.providence.org/locations-directory/s/sacred-heart-medical-center> |
| 295 | The US | Swedish First Hill Campus | 1910 | Seattle | Washington | <https://www.swedish.org/locations/first-hill-campus#axzz2lIu9Xz7q> |
| 296 | The US | Aurora Saint Luke's Medical Center | 1958 | Milwaukee | Wisconsin | <https://www.aurorahealthcare.org/locations/hospital/aurora-st-lukes-medical-center> |
| 297 | The US | Froedtert Hospital | 1980 | Milwaukee | Wisconsin | <https://www.froedtert.com/locations/hospital/froedtert-hospital> |
| 298 | The US | University Hospital | 1924 | Madison | Wisconsin | <https://www.uwhealth.org/findadoctor/location/170> |
| 299 | The US | Charleston Area Medical Center General Hospital | 1984 | Charleston | West Virginia | [www.camc.org/gh](http://www.camc.org/gh) |
| 300 | The US | Ruby Memorial Hospital | NA | Morgantown | West Virginia | <https://wvumedicine.org/ruby-memorial-hospital/> |
